# Abundance and Diversity of Crypto- and Necto-Benthic Coastal Fish Are Higher in Marine Forests than in Structurally Less Complex Macroalgal Assemblages

**DOI:** 10.1371/journal.pone.0164121

**Published:** 2016-10-19

**Authors:** Pierre D. Thiriet, Antonio Di Franco, Adrien Cheminée, Paolo Guidetti, Olivier Bianchimani, Solène Basthard-Bogain, Jean-Michel Cottalorda, Hazel Arceo, Joan Moranta, Pierre Lejeune, Patrice Francour, Luisa Mangialajo

**Affiliations:** 1 Université Nice Sophia Antipolis, CNRS, FRE 3729 ECOMERS, Parc Valrose 28, Nice, France; 2 Muséum National d'Histoire Naturelle, UMR 7208 BOREA, Station Marine de Dinard—CRESCO, Dinard, France; 3 CRIOBE, USR 3278 CNRS-EPHE-UPVD, Perpignan, France; 4 UMR 5110 CNRS/UPVD—CEFREM, Université de Perpignan Via Domitia, Perpignan, France; 5 Septentrion Environnement, Marseille, France; 6 Marine Science Institute, University of the Philippines, Diliman, Quezon City, Philippines; 7 Estación de Investigación Jaume Ferrer, Maó, Menorca, Illes Balears, España; 8 Instituto Español de Ocenografía (IEO), Centre Oceanogràfic de les Balears, Moll de Ponent s/n, Palma de Mallorca, Spain; 9 STARESO, Station de Recherches Océanographiques et Sous-Marines, Calvi, France; 10 Sorbonne Universités, UPMC Univ. Paris 06, INSU-CNRS, UMR 7093, Laboratoire d’Océanographie de Villefranche, Villefranche sur mer, France; Department of Agriculture and Water Resources, AUSTRALIA

## Abstract

In Mediterranean subtidal rocky reefs, *Cystoseira* spp. (Phaeophyceae) form dense canopies up to 1 m high. Such habitats, called ‘*Cystoseira* forests’, are regressing across the entire Mediterranean Sea due to multiple anthropogenic stressors, as are other large brown algae forests worldwide. *Cystoseira* forests are being replaced by structurally less complex habitats, but little information is available regarding the potential difference in the structure and composition of fish assemblages between these habitats. To fill this void, we compared necto-benthic (NB) and crypto-benthic (CB) fish assemblage structures between *Cystoseira* forests and two habitats usually replacing the forests (turf and barren), in two sampling regions (Corsica and Menorca). We sampled NB fish using Underwater Visual Census (UVC) and CB fish using Enclosed Anaesthetic Station Vacuuming (EASV), since UVC is known to underestimate the diversity and density of the ‘hard to spot’ CB fish. We found that both taxonomic diversity and total density of NB and CB fish were highest in *Cystoseira* forests and lowest in barrens, while turfs, that could be sampled only at Menorca, showed intermediate values. Conversely, total biomass of NB and CB fish did not differ between habitats because the larger average size of fish in barrens (and turfs) compensated for their lower densities. The NB families Labridae and Serranidae, and the CB families Blenniidae, Cliniidae, Gobiidae, Trypterigiidae and Scorpaenidae, were more abundant in forests. The NB taxa *Diplodus* spp. and *Thalassoma pavo* were more abundant in barrens. Our study highlights the importance of using EASV for sampling CB fish, and shows that *Cystoseira* forests support rich and diversified fish assemblages. This evidence suggests that the ongoing loss of *Cystoseira* forests may impair coastal fish assemblages and related goods and services to humans, and stresses the need to implement strategies for the successful conservation and/or recovery of marine forests.

## Introduction

Habitat degradation, including the loss of structural complexity (*e*.*g*. loss of structural components such as boulders, trees or corals) [1], is recognized as a major threat to terrestrial, aquatic and marine ecosystems [2, 3]. This may affect ecological processes underlying abundances and distributions of organisms, community structures, ecosystem functions and ecosystem resistance and resilience. Ultimately, this may reduce the potential of the ecosystem to sustainably provide goods and services to humans [3–5].

In temperate subtidal seascapes worldwide, some macrophytes (seaweeds and seagrasses) may form structurally complex benthic habitats, such as kelp forests on hard bottoms and seagrass meadows on soft bottoms. These macrophyte-formed habitats are usually characterized by high biodiversity and high production rates [6]. However, these habitats are being degraded or lost worldwide due to a broad spectrum of anthropogenic and natural causes [7, 8]. This process has negative impacts on associated communities [9], including species that are of ecological and socio-economic importance, such as some fish [10, 11].

**Fig 1:**
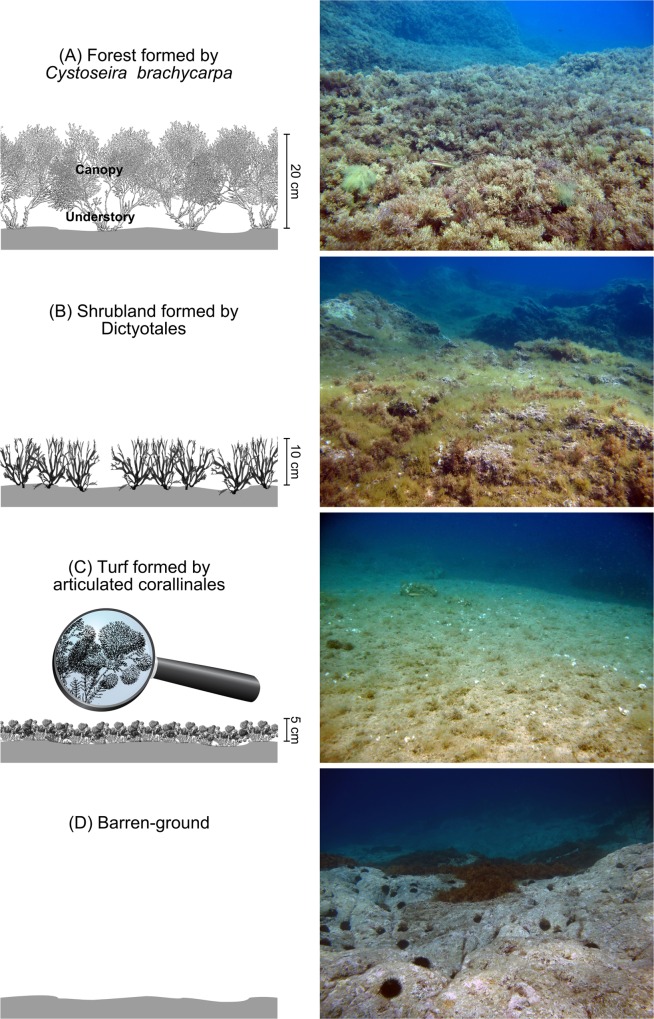
Four habitat types in North-Western Mediterranean subtidal rocky reefs. (A) forest formed by the locally threatened species *Cystoseira brachycarpa* var. *balearica*, and 3 habitat types that may replace lost *Cystoseira* forests: (B) shrubs formed by Dictyotales and Sphacelariales, (C) turf formed by articulated corallinales, and (D) barren characterized by the absence of erect macrophytes. Upper panel: schematic representations of the habitat structure provided by the dominant macrophytes. Habitat complexity decreases from A to D. Lower panel: pictures taken in Corsica during summer 2011, at 8 m depth. Foregrounds span around 2 m width. Modified from Thiriet *et al*. [30].

Mediterranean algal forests are formed in subtidal rocky reefs by *Cystoseira* (and some *Sargassum*) species (Phaeophyceae), forming a dense canopy up to 1 m high (depending on the species, site and season, *e*.*g*. [12, 13–15]). These habitats are suffering degradation as well [16], and past and ongoing losses of *Cystoseira* forests have been recorded throughout the Mediterranean Sea [17, 18–21]. Depending on the identity and intensity of natural and/or anthropogenic stressors, *Cystoseira* forests can be replaced by structurally less complex macroalgal habitats. For instance, in some areas with degraded water quality (*e*.*g*. eutrophication, increased turbidity, waste water discharge, other pollutants), *Cystoseira* forests can be replaced by turfs [22, 23] or shrubland-like habitats (hereafter ‘shrubs’), formed by Dictyotales, Sphacelariales and/or articulated Corallinales [24, 25]. In addition, herbivory can be a major cause of *Cystoseira* forest loss. For example, in areas where sea urchins are abundant (due to natural [26] and/or anthropogenic [27–29] stressors), they can over-graze erect macrophyte assemblages (including *Cystoseira* forests) and produce barren grounds, *i*.*e*. bare rocks covered only by encrusting corallinales, hereafter called 'barrens' [19, 26–29] (Fig 1).

Although the impact of kelp forest loss on coastal ecosystems worldwide is well known [6, 31], effects of Mediterranean *Cystoseira* forest losses on the associated assemblages remain poorly understood. This is mainly because 1) time series analyses are usually not feasible due to a general lack of historical data on Mediterranean subtidal rocky reef ecosystem structures (but see [19]), and 2) *Cystoseira* forest large-scale removal experiments may not be acceptable from a conservation point of view since the recovery of the *Cystoseira* forest would be slow (> 10 years) or even null [18, 32]. Using the ‘space for time’ approach therefore appears a likely solution to gain insights into the possible effects of Mediterranean *Cystoseira* forest losses although it cannot control all the alternative hypotheses that may explain the results obtained through this approach.

Sala *et al*. [33] compared fish assemblage structure within and outside marine reserves in rocky habitats (8–12 m deep, in different algal assemblages) throughout the Mediterranean Sea. The authors identified 4 main ecosystem states. These included one ‘predator dominated’ state with high fish biomass and extensive shrubs, occurring inside the well-enforced marine reserves which prohibit fishing of sea urchin predators, and 3 states occurring outside the well-enforced marine reserves which were poorly protected or unprotected. The authors expected *Cystoseira* forest to be indicative of ‘healthy’ rocky reefs and to be associated with high fish biomasses in well-enforced marine reserves. However, most of the *Cystoseira* forests were found in unprotected (fished) localities and therefore fish biomass in forests was lower than that recorded in well-protected (unfished) localities, generally characterized by shrubs.

A few other studies [34–36] have compared fish assemblage structure between *Cystoseira* forest and other habitats. Despite potential biases related to possible confounding effects from variability in abiotic features known to affect fish assemblages (*e*.*g*. depth, substrate nature and rugosity [37–39]), results suggested the importance of *Cystoseira* forests for some fish taxa, at least for some life stages. One study [40] resolved confounding effects by comparing juvenile fish assemblage structure between patches of *Cystoseira* forests and patches of shrubs sharing the same abiotic features within the same localities/protection levels, and highlighted that juvenile *Symphodus* spp. densities were higher in patches of *Cystoseira* forests while juvenile *Coris julis* densities were lower, and juveniles of all other fish taxa showed no significant difference between habitats.

The above-mentioned studies estimated fish assemblage structure using Underwater fish Visual Census (UVC). UVC has the main advantage of being non-destructive, and is particularly suitable for assessing necto-benthic (NB) fish, which are conspicuous fish swimming just above the substrate [41]. However, UVC underestimates richness and densities of crypto-benthic (CB) fish (*e*.*g*. Blenniidae and Gobiidae), which are 'hard to spot' due to some morphological (small body-size and/or camouflage) and/or behavioural traits (motionless and/or hiding within shelter) [42–45]. Consequently, the sole use of UVC may result in an incomplete picture of fish assemblage composition and density patterns biased towards conspicuous NB fish. CB fish assemblage structure can reliably be assessed only by using harvesting methods (*e*.*g*. using anesthetic such as quinaldine or piscicide such as rotenone: [42, 43–48]).

Kovačić *et al*. [47], for the first time in the Mediterranean Sea, used a quantitative harvesting method specifically designed to sample CB fish by using quinaldine within a 1 m² sampling area. This enabled sampling of a higher number of CB species compared to previous studies using UVC [49, 50], and assessment of CB fish densities, which is an improvement on previous qualitative harvesting methods (*e*.*g*. [51]). Thus, Kovačić *et al*. [47] highlighted the high diversity and densities of CB fish inhabiting various benthic habitat types (from 1 to 20 m depth, in the Adriatic Sea). Unfortunately, this study [47] and the previous ones on CB fish [49–51] did not include *Cystoseira* forests. CB fish assemblages associated with *Cystoseira* forests remain therefore mostly unknown, although they may have important roles in ecosystem functioning [46].

To fill this gap and to assess the potential role of the *Cystoseira* forest for fish assemblages, we carried out a spatial comparison of small-medium (total length < 30 cm) fish assemblage structure between *Cystoseira* forest and two structurally less complex habitat types usually replacing the forests (turf and barren). In each of the 3 habitat types investigated, we sampled NB fish using UVC, and for the first time, CB fish using Enclosed Anaesthetic Station Vacuuming (EASV).

## Material and Methods

### Sampling design

**Fig 2:**
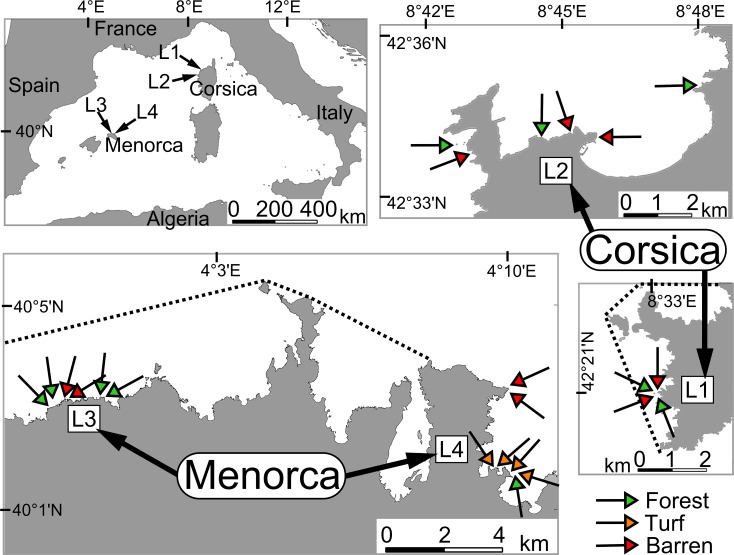
Location of the sampling sites. Green filled arrows indicate forest sites, red filled arrows indicate barren sites, orange filled arrows indicate turf sites. Localities 'L1' and 'L3' were within the Marine Protected Areas (MPA) Scandola Marine Reserve and Norte de Menorca Marine Reserve, respectively. Dotted lines indicate MPA boundaries. Localities 'L2' and 'L4' were both outside MPAs. See also S2 Table for geographical coordinates of all sites. Public domain source of backgrounds maps: OpenStreetMap contributors, available under ODbL licence at http://www.openstreetmap.org/. Figure modified from Thiriet *et al*. [30].

We sampled fish and macrophyte assemblages within 2 regions of the North-Western Mediterranean Sea: Corsica (10 sites, May 2011) and Menorca (13 sites, July 2011). For logistical reasons, we did not sample the two regions at the same time. However, we sampled both regions during the period of maximum temporal stability of macrophyte biomass (late spring to early summer [15]) to minimize the potential effects of variation in habitat structures, which may have impacted fish assemblages. Within each of the two region-time combinations (Corsica-May and Menorca-July), we sampled two localities (L): one protected (within a marine protected area, L1 and L3 respectively in Corsica and Menorca) and one unprotected (outside marine protected area, L2 and L4 respectively in Corsica and Menorca) (Fig 2). We aimed to sample all habitat types (forest, shrub, turf and barren) within each locality in order to avoid possible confounding effects between the putative effects of habitat types and inter-locality variations related to natural variations and/or potential protection effects (which are at present not distinguishable [52]). Within each locality, we found 1 to 4 sampling sites (750 to 1000 m² areas) of both forest and barren. Turf was only sampled at 4 sites in Menorca (within L4). We did not find suitable areas for sampling shrub (Fig 2, and S2 Table for geographical coordinates of all sites). This was due to our stringent procedure of sampling site selection, which was as follows. Within each locality, sampling sites were randomly chosen among the 750 to 1000 m² areas that fulfilled two criteria: (1) at least 80% of the area was covered by one of the 4 targeted habitat types, and (2) the whole area was between 4 m to 9 m in depth, presenting only monolithic rock (as opposed to blocks, pebbles etc.), with gentle slope (0° to 15°) and low substrate rugosity (*i*.*e*. holes, steps, crevasses and overhangs were avoided). These abiotic features known to affect fish assemblage structure [39] were constrained in order to avoid possible confounding effects. The surface area of sampling sites (750 to 1000 m²) was chosen as a trade-off between (1) a surface area small enough so that it was possible to find sufficient areas fulfilling all of the above criteria for each habitat type within each locality, and (2) a surface area large enough so that it may be regarded as a habitat rather than a patch, at least for low mobility organisms (see below).

### Data collection

#### Ethics statement

Small surfaces (625 cm²) of macrophyte communities were harvested (using chisel and hammer) to perform species identification and biomass assessment in the laboratory. Removal of algae was necessary since non-destructive methods (*e*.*g*. visual estimation of percent cover) did not allow for quantification of the understory macrophyte assemblages. Even though knowledge regarding the role of *Cystoseira* species in coastal ecosystems is improving, as is the awareness that they may be locally threatened (for this reason most *Cystoseira* species are listed in the Bern Convention and in the Aspim Protocol), no conservation measures have yet been adopted at the national or international level. Independently of regulations, the surface area sampled and the number of samples were kept to the minimum. Care was taken to avoid sampling isolated populations or damaged / declining forests.

The non-destructive methodology UVC was used to gather data on NB fish. However, UVC is not suitable for gathering data on CB fish. Thus, we harvested CB fish by scuba diving using EASV (see below). Fish were anesthetized before being collected by spraying locally (1 m²) 2 L of 5 ppm quinaldine solution (0.01 L of quinaldine, 0.1 L of acetone and 1.89 L of seawater). After each dive, collected fish, still anesthetized, were killed immediately by anaesthesia overdose (immersion in a 2 L tank filled with 25 ppm quinaldine solution: 0.05 L of quinaldine, 0.5 L of acetone and 1.45 L of seawater), following Directive 2010/63/EU of the European Parliament and of the Council on the protection of animals used for scientific purposes. The EASV protocol did not require animal ethics committee approval since fish were killed in the field directly after collection (no housing, husbandry nor experiments) using anaesthesia overdose. No negative effects of EASV on the benthic community were recorded during the sampling. A few mobile macroinvertebrates were caught unintentionally on rare occasions. They were released alive and unharmed after each dive. Sessile benthic organisms did not show any damage related to the EASV sampling procedure. The whole experimental protocol was approved by the relevant regulatory bodies of each sampling locality. Permission for sampling in Locality 1 (Corsica, inside the MPA Réserve Naturelle de Scandola) and Locality 2 (Corsica, unprotected) was issued by the Direction Interrégionale de la Mer Méditerranée in the form of the prefectural ruling *Décision du 8 avril 2011*. Additional permission for sampling in Locality 1 (inside MPA) was issued by the Parc Naturel Regional de Corse (the institution managing the MPA). The permission for sampling in Locality 3 (Menorca, inside the MPA Reserva del Nord de Menorca) and Locality 4 (Menorca, unprotected) was issued by the Direccio General de Pesca, Govern de Illes Balears (the Spanish administration in charge of Maritime affairs in Menorca, managing the MPA).

#### Macrophyte Assemblage

We measured biomass of macrophytes in order to verify *a posteriori* that sampling sites (visually selected) were appropriately classified into meaningful and objective habitat types, and to describe the macrophyte assemblages. While scuba diving, we scraped (using chisel and hammer) all non-encrusting macrophytes in three replicate 25 x 25 cm² quadrats at each site. Each sample was placed in an individual zip-lock bag. After the dive, macrophyte samples were individually removed from their bags, wrapped in a terrycloth soaked with 70% alcohol, packed again in a hermetic bag and stored in a cooler until we reached the field laboratory where we stored samples in a freezer. Macrophyte biomass was measured within 3 days after collection. Excess water and alcohol were removed from samples by centrifuging them using a salad spinner for 30 seconds [33]. Samples were individually sorted and weighed using operational taxonomic units (S1 Table). In order to characterize fish habitat types, macrophytes were pooled into 6 functional groups before data analyses: (1) canopy-forming macrophytes (mostly *Cystoseira brachycarpa* var. *balearica*, with sometimes less than 5% of *C*. *compressa* and/or *Sargassum* spp.), (2) large erect macrophytes (*e*.*g*. *Dictyota* spp.), (3) small erect macrophytes (*e*.*g*. *Acetabularia acetabulum*), (4) turf-forming articulated corallinales, (5) turf-forming filamentous macrophytes, and (6) massive macrophytes (*i*.*e*. *Codium bursa*) (S1 Table).

#### Fish assemblage

Given the extent of our sampling sites (750 to 1000 m²), we did not take into account some NB fish that clearly move on broader spatial scales, such as transient predators (*e*.*g*. *Dentex dentex*), shoaling species (*e*.*g*. *Chromis chromis*, *Oblada melanura*, *Sarpa salpa*) and also large (Total Length, TL > 30 cm) resident fish (*e*.*g*. (sub-) adult *Epinephelus marginatus*, large-sized *Diplodus* spp.). We restricted our fish surveys to fish individuals that were a priori more sedentary at the scale of our sampling sites, hereafter referred as ‘small-medium resident fish’, which were juveniles of all CB and NB fish species, along with all older life stages for the small-medium species (maximum TL <30 cm) or only some of the older stages for larger species, depending on their maximum TL. Hence, in the present study, ‘all fish’ refers only to all individuals of small-medium resident fish. Likewise, ‘total density’ and ‘total biomass’ also refer to small-medium resident fish.

**Fig 3:**
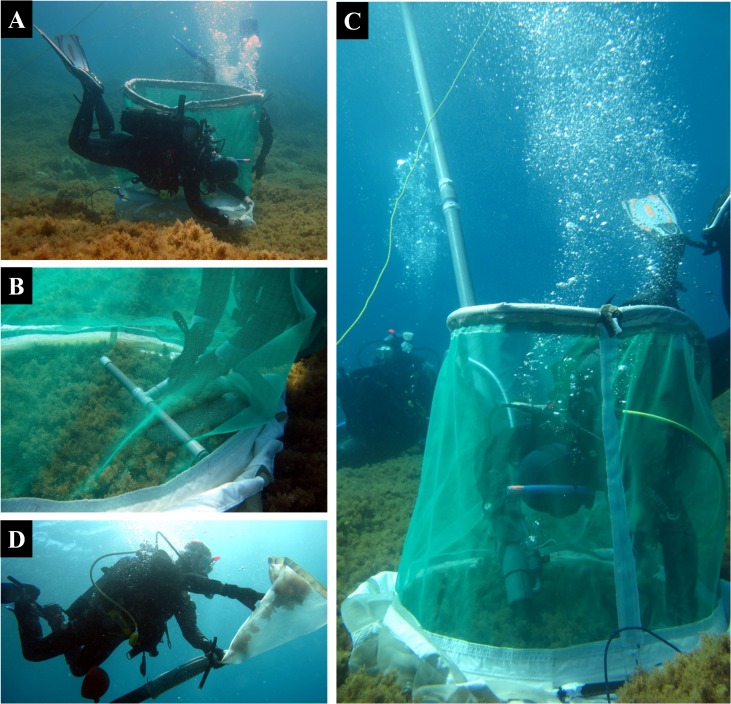
Quantitative sampling of crypto-benthic fish using Enclosed Anaesthetic Station Vacuuming. Steps include: (A) Setting-up the perimeter fence by arriving vertically from 2 m above the substrate, and moulding the base of the perimeter fence (weighted with galvanized chain) to the substrate in order to avoid fish escapes; (B) Spraying of the anaesthetic and waiting for 1 minute; (C) Vacuuming for 2 minutes using an air-lift sampler; (D) Closing the collecting bag as soon as the vacuuming session ends. Modified from Thiriet *et al*. [30].

We used EASV to sample CB fish, which were defined in the present study as the 'hard to spot' fish individuals (see [47] for other definitions), including (1) early juveniles of NB species, which are small-sized individuals (TL < 25 mm for Labridae, < 35 mm for Serranidae) spending most of their time hidden within macrophytes [53], and (2) all life stages of CB fish species (*e*.*g*. Blenniidae and Gobiidae). During daylight (10AM–4PM), we conducted 3 replicate EASV samples of 1 m² at each site. The 1 m² sample area was enclosed by a perimeter fence and all fish inside were collected using anaesthetic and an air-lift pump (Fig 3). The perimeter fence (0.56 m in radius, 1 m in height) was a circular 1 mm nylon mesh mounted on a metal hoop. The base of the perimeter fence was extended by a tissue strip (0.25 m in width) weighted with galvanized chain so that the base of the perimeter fence could be moulded to the substrate shape. Two litres of anaesthetic solution (5 ppm quinaldine solution: 1 cl of quinaldine, 10 cl of acetone, 189 cl of seawater) were sprayed 15 cm above the substrate [47]. One minute later, fish were collected by vacuuming using an air-lift pump (with a 1mm mesh collecting bag). The pump head was moved all around the 1 m² sample area for 2 minutes. After the dive, fish samples, still anesthetised, were killed by an anaesthetic overdose and stored in plastic tubes filled with 70% alcohol. In the laboratory, samples were sorted, and individuals were measured (to the nearest mm), weighed (mg), and identified to the species level whenever possible, or alternatively to the family level. EASV samples contained both CB and NB fish individuals. NB fish were removed in order to prevent overlap with NB sampled using UVC.

We used UVC for sampling small-medium resident NB fish, which are late juveniles of NB species (≥ 25 mm for Labridae, ≥ 35 mm for other taxa) that reached the NB behaviour stage [53] and (sub-) adult fish individuals (TL <30 cm) belonging to NB species, hereafter referred as ‘NB fish’. We did not use the standard method 5 x 25 m² transect [41] because it would have not been possible to fit multiple 125 m² replicates within our sampling sites (750 to 1000 m²) and to meet the independence assumption. Instead, we used 9-m² stationary-point snapshot-count, conducted during daylight (10AM–4PM). Six random replicates, which were at least 10 m apart from each other, were done at each site. The 9-m² sampling area was a semicircle 2.5 m in radius in front of the diver, excluding the inner semicircle 0.7 m in radius nearest to the diver (S1 Fig). The diver did a snapshot count of every NB fish individual inside the sampling area at the time the census started, by estimating the species and the body size (total length to the nearest 0.5 cm for fish ≤ 5cm, to the nearest 1 cm for larger fish). Fish biomass was estimated using the existing length—weight relationship from the literature [54, 55].

### Data analyses

#### Data pre-processing

In order to analyse relationships between macrophytes and NB and CB fish, data for algae, CB and NB fish (each stored in a database) were aggregated at the site level, which was the smallest sampling unit shared by the 3 databases. Biomass (and/or densities) was averaged over replicates and mean values (for each site) were stated in grams (and/or number of individuals) per 1 m² for macrophytes and per 10 m² for fish. These values were used for all statistical analyses. Using sites as statistical units did not lower the power of the analyses of variances (see below) when comparing inter-habitat variability over intra-habitat (inter-site within habitat) variability [56].

#### Habitat types

In order to verify *a posteriori* that sampled sites were appropriately classified into meaningful and objective habitat types (forest, turf, or barren), biomass of the 6 macrophyte functional groups was used for clustering sites into internally homogenous groups of habitat types, by running the PRIMER routine combining hierarchical clustering (group-average) and Type 1 SIMPROF test (defining the most appropriate number of clusters), on Bray-Curtis dissimilarity matrices with square root transformed data [57, 58].

#### Fish assemblages

To compare multiple aspects of fish assemblage structure between habitat types, we considered 9 multivariate descriptors, combining 3 sets of fish category (only CB fish sampled by EASV, only NB fish sampled by UVC and all fish) and 3 types of metrics (presence/absence, density and biomass). Jaccard similarity was used on presence/absence, and Bray-Curtis dissimilarity was used on square root transformed densities and biomasses. Similarly, nine univariate descriptors were also used: number of taxa, total density and total biomass for each of the 3 sets of fish category.

Based on each descriptor (multivariate or univariate), we tested for putative differences between forest and barren, by using 3-way permutational (multivariate or univariate) analyses of variance (PERMANOVAs): factor region-time ('RT', fixed, 2 levels: Corsica-May and Menorca-July), factor locality-protection ('LP', fixed, 2 levels nested within each 'RT' level), factor habitat ('HA', fixed, 2 levels: forest, barren). The habitat turf was excluded from the PERMANOVA design because turf sites were sampled only within locality L4 of Menorca-July, this would have induced a large amount of empty cells in the design. Because the design was still unbalanced, we used Type III sum of squares (SS). P-values were obtained by 9999 permutations of residuals under a reduced model. Post-hoc pair-wise comparisons were used when appropriate. Univariate PERMANOVA were based on Euclidean distances which makes this a non-parametric test that is equivalent to a parametric ANOVA but free from the assumption of normality of residuals [59].

To identify groups of fish taxa responding similarly to factors evidenced as significant by PERMANOVA on densities of all fish, we performed Type 2 and Type 3 SIMPROF tests. Densities were averaged for each level of the factor combining all significant factors (*i*.*e*. the combination of habitat and region-time, see ‘Results‘). Type 2 SIMPROF test tested the null hypothesis of 'no associations among taxa'. Type 3 SIMPROF test was used to identify statistically distinct groups of taxa, by combining hierarchical clustering (group-average) of taxa and Type 2 SIMPROF test (see [58] for more details). Only taxa that occurred in at least 4 out of the 23 sampling sites were retained since the method is sensitive to the inclusion of the rarest taxa [58]. For this test (very conservative since it controls experiment-wise type I error rate [58]), we used the threshold 0.1 as significance level instead of the common threshold 0.05, since we aimed to explore ecological trends rather than to test ecological inferences. For all other statistical significance tests (of inferences), we used 0.05 as threshold.

To visualize multivariate patterns, Principal Coordinates Analyses (PCoA) were used on the 3 dissimilarity matrices involving all fish taxa. Due to high variability among replicates that prevented getting reliable visualizations of dissimilarities in 2D (first two PCoA axes), we used only centroids of each level of the factor combining all factors (HA x RT x LP(RT)).

All SIMPROF tests and PERMANOVAs were performed using the PRIMER 6 and PERMANOVA + B20 package [60, 61]. All graphical visualizations were performed in R Environment [62] using the libraries vegan [63] and ggplot2 [64].

As a supplement, we investigated fish body-size (total length) distributions in order to (1) assess the relative contributions of CB and NB fish to total fish density and total fish biomass, and (2) visualize putative differences among habitats. Methods and results of this complementary analysis are reported in S1 Text and S2 Fig.

## Results

### Habitat types

**Fig 4:**
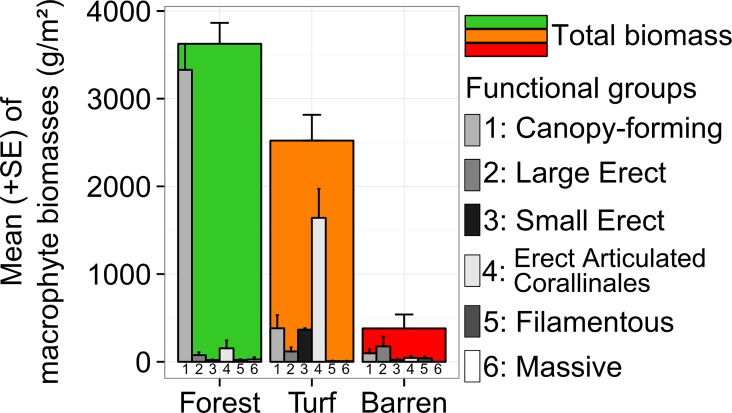
Macrophyte assemblage structures discriminating the 3 habitat types. Mean total macrophyte biomass (+SE) and mean biomass (+SE) of the 6 macrophyte functional groups for each of the 3 habitat types sampled (see also Fig 1A, 1C and 1D). Modified from Thiriet *et al*. [30].

Biomass of the 6 macrophyte functional groups was not homogeneous among sites (Type 1 SIMPROF test, π = 2.186, p = 0.02). The combined clustering/SIMPROF analysis showed that 3 groups of sites were significantly different from each other but internally homogeneous. The clustering matched with our *a priori* grouping of sampling sites by habitat types (presented in Fig 2). The habitat forest exhibited the highest total macrophyte biomass (Fig 4). It was dominated by Fucales (> 90% of *Cystoseira brachycarpa* var. *balearica* in both Corsica and Menorca) forming a dense canopy (around 15 to 20 cm in height). The habitat turf exhibited lower total macrophyte biomass (70% of forest's biomass). It was dominated by erect articulated Corallinales forming a dense layer of turf (around 5 cm thick). The turf layer sometimes smothered short-sized individuals of Fucales and/or was sparsely epiphyted by some other erect macrophytes. The habitat barren exhibited very low total macrophyte biomass (10% of forest's biomass). In some barren sites, short-sized individuals of Fucales (< 5cm) and/or of erect non canopy-forming macrophyte (mostly *Padina* sp.) were sparsely present.

### Multivariate descriptors of fish assemblage structure

All 9 multivariate descriptors considered were significantly different between forest and barren, and between Corsica-May and Menorca-July (Table 1). This showed that (1) the differences in the whole fish assemblage structure were due to both the subsets of CB and NB fish, and (2) the differences in fish assemblage structure were in terms of taxa composition, and possibly also in terms of densities and biomass.

**Table 1:** Results of multivariate PERMANOVAs comparing fish assemblage structure between forest and barren.

			All fish			Crypto-benthic fish			Necto-benthic fish		
Data and dissimilarity measure used	Source	df	SS	F		SS	F		SS	F	
Jaccard on presence / absence	HA	1	7334.8	**5.17**	***	4254.2	**3.50**	*	9762.7	**8.06**	***
	RT	1	7428.1	**5.23**	***	6140.4	**5.06**	**	7296.3	**6.02**	***
	LP(RT)	2	3296.0	1.16	^ns^	1022.6	0.42	^ns^	3809.2	1.57	^ns^
	RTxHA	1	2056.1	1.45	^ns^	2626.2	2.16	°	997.2	0.82	^ns^
	LP(RT)xHA	2	3570.9	1.26	^ns^	1508.7	0.62	^ns^	4356.3	1.80	°
	Residual	11	15613.0			13359.0			13332.0		
Bray-Curtis on square root transformed densities	HA	1	5139.7	**4.08**	**	4389.9	**4.14**	*	8452.9	**10.36**	***
	RT	1	7498.9	**5.96**	***	7209.2	**6.81**	***	5860.0	**7.18**	***
	LP(RT)	2	3023.3	1.20	^ns^	1076.5	0.51	^ns^	3456.5	2.12	°
	RTxHA	1	2872.2	2.28	°	2369.0	2.24	°	1598.7	1.96	^ns^
	LP(RT)xHA	2	3177.1	1.26	^ns^	1354.1	0.64	^ns^	3640.2	2.23	°
	Residual	11	13849.0			11652.0			8977.9		
Bray-Curtis on square root transformed biomasses	HA	1	5621.5	**5.75**	***	6097.4	**2.99**	*	5629.7	**6.10**	***
	RT	1	6475.2	**6.62**	***	5491.8	**2.70**	*	6576.4	**7.13**	***
	LP(RT)	2	5776.4	**2.96**	**	3036.8	0.75	^ns^	5685.4	**3.08**	***
	RTxHA	1	2272.3	2.32	°	3810.9	1.87	°	2234.4	2.42	°
	LP(RT)xHA	2	4075.9	2.09	°	2987.4	0.73	^ns^	4184.4	2.27	°
	Residual	11	10751.0			22401.0			10149.0		

HA: habitat; RT: region-time; LP: locality-protection.

^ns^ not significant

° p < 0.1

* p < 0.05

** p < 0.01

*** p < 0.001.

**Fig 5:**
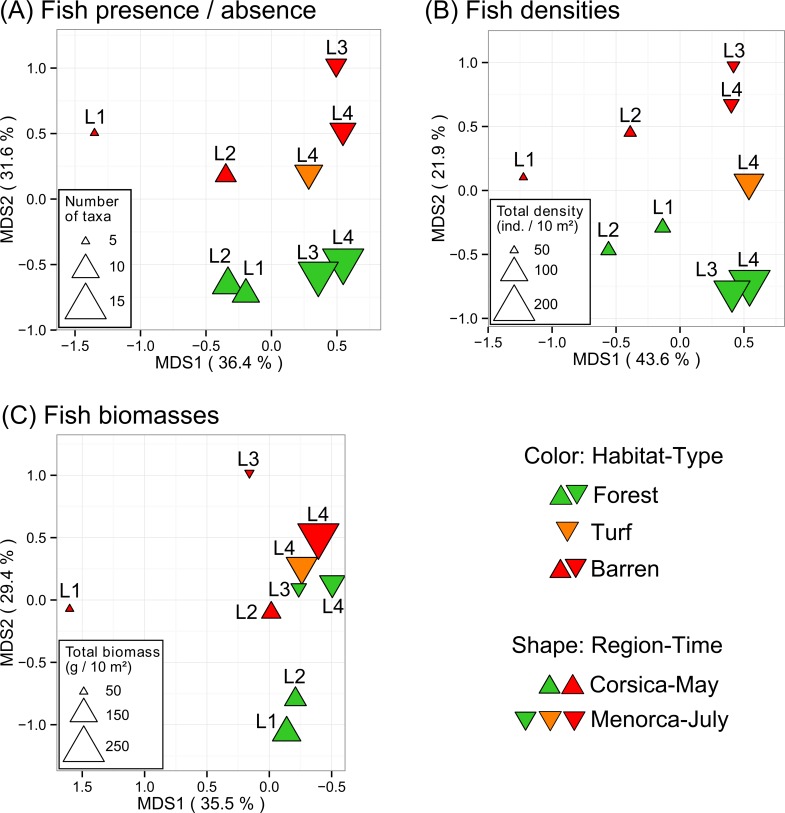
**Fish assemblage structure compared among habitats and regions-times, in terms of (A) presence / absence, (B) densities, and (C) biomasses of all crypto- and necto- benthic fish.** Principal coordinates analyses (PCoA) were built using dissimilarities among centroids of each levels of the combined factor habitat X locality-protection (region-time), which were computed using Jaccard dissimilarity for presence / absence data and Bray-Curtis dissimilarity for both square root transformed densities and biomass data. First two axes (MDS 1 and 2) are plotted and percentages of explained variance are indicated within brackets. Labels refer to the 4 locality-protection levels (see Fig 2). Modified from Thiriet *et al*. [30].

When considering fish assemblage composition (presence/absence of all fish), the inter-habitat and inter- region-time differences were additive (Table 1 and Fig 5A). When considering fish densities, inter-habitat differences appeared slightly higher within Menorca-July than within Corsica-May (Table 1 and Fig 5B). In contrast, when considering fish biomass, inter-habitat differences appeared slightly lower within Menorca-July than within Corsica-May (Table 1 and Fig 5C).

Considering the habitat turf (not included in PERMANOVAs, see Material & Methods section) sampled only within Locality 4 (L4), the centroid of turf X L4 was positioned between the centroids of barren X L4 and forest X L4, particularly on PCoA biplot based on presence/absence (Fig 5A) and densities (Fig 5B).

### Univariate descriptors of fish assemblage structure

**Fig 6:**
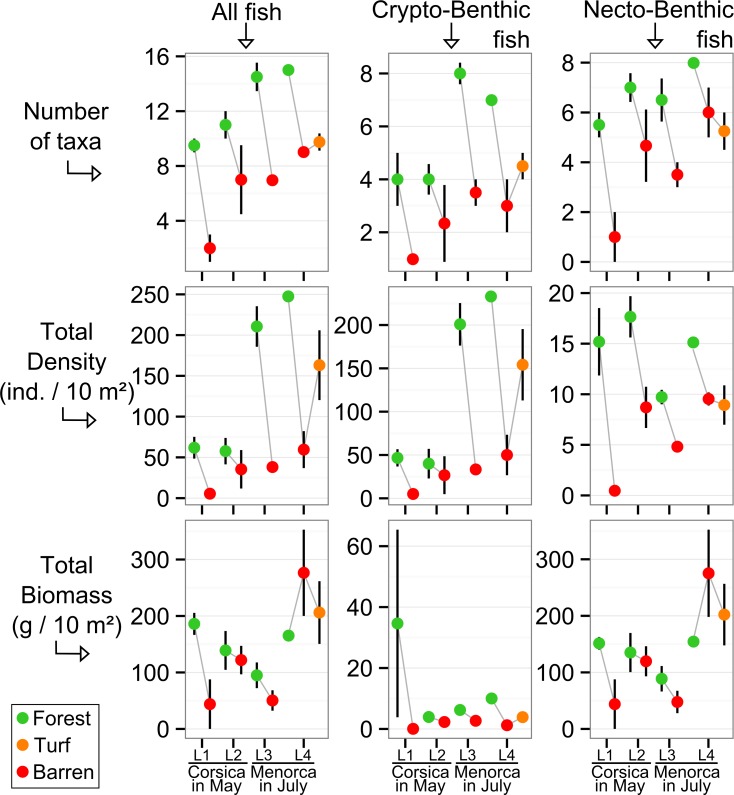
Univariate descriptors of fish assemblage structure compared among habitats and regions-times. Mean values (+/- SE) of the number of taxa (observed per site), the total density and the total biomass of all fish, only crypto-benthic fish and only necto-benthic fish, for each habitat x locality-protection (region-time) level combination. Modified from Thiriet *et al*. [30].

The number of taxa of all fish, of the subset CB fish and of the subset NB fish were all significantly higher in forest than in barren (Table 2 and Fig 6). The number of taxa of all fish and of the subset CB fish were both significantly higher in Menorca-July than in Corsica-May (Table 2 and Fig 6). The number of taxa of all fish, of the subset CB fish and of the subset NB fish, were similar between the turf sites and the barren sites of the same locality (see L4 in Fig 6).

**Table 2:** Results of univariate PERMANOVAs comparing fish assemblage structure between forest and barren.

			All fish			Crypto-benthic fish			Necto-benthic fish		
Response variable	Source	df	SS	F		SS	F		SS	F	
**Number of taxa**	HA	1	159.6	**29.50**	***	44.3	**23.00**	***	35.8	**13.72**	**
	RT	1	65.4	**12.08**	**	26.4	**13.72**	**	8.7	3.33	°
	LP(RT)	2	28.1	2.60	^ns^	2.1	0.54	^ns^	23.1	**4.44**	*
	RTxHA	1	1.0	0.19	^ns^	3.8	1.95	^ns^	0.9	0.33	^ns^
	LP(RT)xHA	2	8.4	0.77	^ns^	1.2	0.31	^ns^	3.3	0.63	^ns^
	Residual	11	59.5			21.2			28.7		
**Total density**	HA	1	492.8	**39.16**	***	420.5	**34.95**	***	2.9	**39.54**	***
	RT	1	399.7	**31.77**	***	406.8	**33.81**	***	0.0	0.43	^ns^
	LP(RT)	2	19.2	0.76	^ns^	12.1	0.50	^ns^	1.1	**7.49**	**
	RTxHA	1	203.0	**16.13**	**	223.5	**18.57**	**	0.5	**6.77**	*
	LP(RT)xHA	2	8.1	0.32	^ns^	5.9	0.25	^ns^	0.2	1.38	^ns^
	Residual	11	138.4			132.7			0.8		
	Pairwise tests Forest *vs* Barren		Corsica: t = 2.05°; Menorca: t = 5.86**			Corsica: t = 1.50^ns^; Menorca: t = **5.70** ***			Corsica: t = 5.27**; Menorca: t = 5.51**		
**Total biomass**	HA	1	21.8	0.70	^ns^	6.0	3.42	^ns^	4.9	0.16	^ns^
	RT	1	23.5	0.76	^ns^	1.1	0.62	^ns^	34.7	1.11	^ns^
	LP(RT)	2	395.3	**6.38**	*	4.9	1.38	^ns^	404.5	**6.48**	*
	RTxHA	1	130.6	4.22	°	1.5	0.82	^ns^	104.6	3.35	°
	LP(RT)xHA	2	202.1	3.26	°	6.6	1.88	^ns^	166.7	2.67	^ns^
	Residual	11	340.7			19.4			343.0		

HA: habitat; RT: region-time; LP: locality-protection.

^ns^ not significant

° p < 0.1

* p < 0.05

** p < 0.01

*** p < 0.001.

The total densities of all fish and of CB fish in Menorca-July were significantly higher in forest than in barren. This trend was almost significant within Corsica-May (Table 2 and Fig 6). The higher densities of CB fish in forests of Menorca-July compared to forests of Corsica-May were mainly driven by very small sized individuals (TLs between 5 and 15 mm) that were highly abundant in Menorca-July (see below description of group 6, and S1 Text and S2 Fig). The total NB fish density was significantly higher in forest than in barren within both regions, but this was more pronounced within Corsica-May (Table 2 and Fig 6). The total densities of (1) all fish and (2) only CB fish recorded in turf were intermediate between densities recorded in barrens (low) and in forests (high) of the same locality (Fig 6). Contrastingly, the total NB fish density recorded in turfs was similar to that recorded in barrens (Fig 6). None of the 3 total biomass variables (all fish, only CB and only NB) showed significant difference between habitat types or between regions-times (Table 2 and Fig 6).

Total CB fish density represented on average 92% of all small-medium resident fish density, and total CB fish biomass represented on average 17% of all small-medium resident fish biomass. This was related to the fact that total-lengths (and correlated body-weight) of CB fish were on average smaller than total-lengths of NB fish (Table 3, S1 Text and S2 Fig).

**Table 3:** Groups of fish sharing the same density variations across habitats and regions-times.

G	Family	Taxa (and life history traits)	Size range in mm	Mean Size (SE)	Mean densities (SE) (indiv./ 10 m²)				
					Corsica-May		Menorca-July		
					Forest (n = 5)	Barren (n = 5)	Forest (n = 5)	Barren (n = 4)	Turf (n = 4)
1	Sparidae	*Diplodus sargus* (NB)	[80,300]	144 (20.2)	0.04 (0.04)	0.3 (0.17)	-	-	0.05 (0.05)
2	Labridae	*Thalassoma pavo* (NB)	[50,160]	92.7 (3.1)	-	-	0.3 (0.15)	2.41 (0.33)	1.16 (0.54)
	Sparidae	*Diplodus vulgaris* (NB)	[60,160]	109.5 (3.4)	0.04 (0.04)	0.11 (0.07)	0.26 (0.26)	1.39 (0.44)	0.65 (0.44)
	Total G2				0.04 (0.04)	0.11 (0.07)	0.56 (0.38)	3.8 (0.77)	1.81 (0.67)
3	Labridae	*Symphodus ocellatus* (NB)	[30,120]	59.8 (1)	7.52 (1.42)	0.19 (0.19)	0.89 (0.26)	-	0.05 (0.05)
4	Labridae	*Symphodus roissali* (NB)	[25,150]	70.6 (3.2)	1.96 (0.4)	-	1.33 (0.34)	-	0.05 (0.05)
	Scorpaenidae		[15,43]	29 (5.1)	1.33 (1.33)	-	1.33 (0.82)	-	0.83 (0.83)
	Serranidae	*Serranus cabrilla* (NBJ)	[12,32]	20 (2.1)	2 (1.33)	1.33 (1.33)	4.67 (0.82)	-	0.83 (0.83)
		*Serranus cabrilla* (NB)	[35,180]	94.9 (6.7)	0.59 (0.27)	0.07 (0.07)	0.63 (0.16)	0.09 (0.05)	0.09 (0.05)
		*Serranus scriba* (NB)	[60,240]	135.3 (6.8)	0.3 (0.17)	0.04 (0.04)	0.52 (0.11)	0.19 (0.13)	0.14 (0.09)
	Tripterygiidae (CBS)		[13,56]	20.2 (1)	12.67 (6.27)	5.33 (3.89)	16 (2.21)	3.33 (2.36)	1.67 (1.67)
	Total G4a				18.85 (6.69)	6.78 (5.18)	24.48 (2.53)	3.61 (2.36)	3.61 (1.29)
5	Labridae	*Coris julis* (NB)	[25,250]	88.9 (1.8)	4.96 (1.19)	4.26 (1.7)	5.89 (1.02)	2.55 (0.62)	5.6 (1.18)
		*Symphodus tinca* (NB)	[35,300]	126.6 (9.3)	1.04 (0.67)	0.41 (0.28)	0.15 (0.07)	0.19 (0.19)	0.32 (0.21)
	Total G4b				6 (1.01)	4.67 (1.93)	6.04 (1)	2.73 (0.75)	5.93 (1.31)
6	Clinidae (CBS)		[10,23]	17.9 (1.7)	-	-	4.67 (1.7)	-	-
	Gobiesocidae (CBS)		[5,14]	8 (0.2)	2.67 (1.94)	0.67 (0.67)	50.67 (13.6)	4.17 (1.6)	8.33 (4.41)
	Labridae	*Coris julis* (NBJ)	[15,21]	18.7 (0.8)	-	-	3.33 (2.58)	-	1.67 (0.96)
		*Symphodus* spp. (NBJ)	[7,20]	10.4 (1.1)	-	-	10 (3.5)	-	1.67 (1.67)
	Total G5				2.67 (1.94)	0.67 (0.67)	68.67 (10.98)	4.17 (1.6)	11.67 (4.19)
7	Blenniidae (CBS)		[15,43]	21.5 (1)	2.67 (1.94)	1.33 (1.33)	14 (4.52)	8.33 (2.15)	15.83 (4.79)
	Gobiidae (CBS)		[7,95]	13.9 (0.6)	10.67 (3.4)	2.67 (1.94)	101.33 (25.55)	25.83 (9.85)	122.5 (35.26)
	Mullidae	*Mullus surmuletus* (NB)	[50,100]	75 (2)	-	-	0.7 (0.32)	0.37 (0.31)	0.69 (0.69)
	Total G6				13.33 (4.94)	4 (2.45)	116.04 (28.85)	34.54 (8.56)	139.03 (39.24)

**Fig 7:**
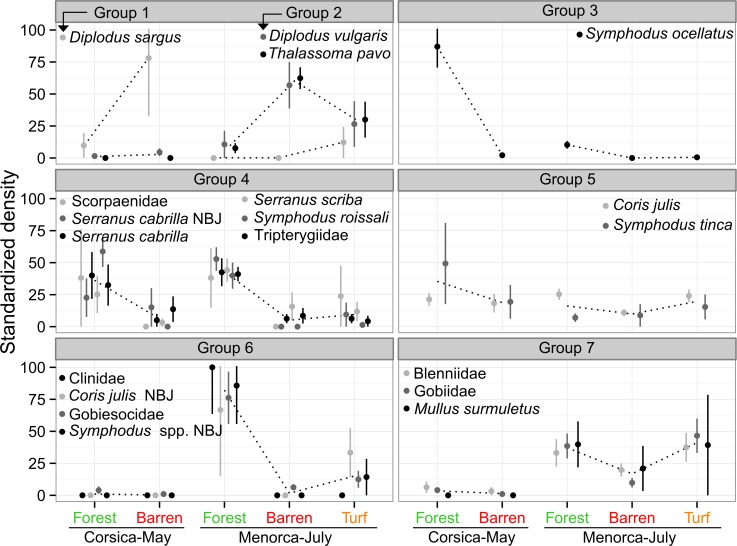
Groups of fish sharing the same density variations across habitats and regions-times. Mean standardized density (+/-SE) indicates variations of every fish taxon on a common scale even if their respective absolute densities may be different. NBJ: early juveniles of necto-benthic species sampled by EASV, while late juveniles and (sub-)adults were sampled by UVC. See Table 3 for detailed information about body-size and absolute densities of each fish taxon. Modified from Thiriet *et al*. [30].

G: groups delimited by Type 3 SIMPROF test (see also Fig 7); NB: late juveniles and (sub-) adult of necto-benthic species sampled by UVC; NBJ: early juveniles of necto-benthic species sampled by EASV; CBS: all life stages of crypto-benthic species sampled by EASV. Size are fish total lengths expressed in mm.

### Groups of fish

The null hypothesis of 'no associations among species' was rejected (Type 2 SIMPROF test, π = 0.043, p = 0.021). Seven groups of taxa were identified as significantly different from each other but internally homogeneous (p-values < 0.1) in terms of their trends in density variations across habitat and regions-times (results of Type 3 SIMPROF tests in Fig 7 and Table 3).

Groups 1 and 2 were composed of NB species that were more abundant in barren but species were segregated by regions-times. *Diplodus sargus* (group 1) was recorded almost only in barren in Corsica-May. *Diplodus vulgaris* and *Thalassoma pavo* (group 2) were recorded almost only in Menorca-July, with higher densities in barren, intermediate in turf, and lower in forest.

Groups 3 to 6 were composed of fish generally more abundant in forest in at least one region-time. The NB *Symphodus ocellatus* (forming group 3) was highly abundant in forests of Corsica-May and was also abundant in forests of Menorca-July, while almost never recorded in barren and turf, irrespective of the region-time.

Group 4 was the larger and more diversified group, composed of both CB and NB fish with sizes ranging from 1 to 24 cm (Table 3), which were abundant in forest but rarely observed in other habitats, consistently across regions-times.

Group 5 was composed of the labrids *Coris julis* and *Symphodus tinca*, which were slightly more abundant in forest (especially for *S*. *tinca*), although densities were high in every habitat (Table 3).

Group 6 was composed essentially of CB juvenile fish, belonging to CB taxa (Clinidae and Gobiesocidae juveniles) and NB taxa (early juveniles of *Coris julis* and *Symphodus* spp. at the CB stage). They were almost exclusively recorded in Menorca-July (excepting Gobiesocidae) where they were more abundant in forest than in turf and almost absent in barren. The few individuals of Gobiesocidae recorded in Corsica-May (relatively to the other region-time, Table 3) were also more abundant in forest.

Group 7 was composed of CB fish belonging to the family Blenniidae and Gobiidae and of juveniles of the NB species *Mullus surmuletus*. They were mainly recorded in Menorca-July, with higher densities in forest and turf than in barren. The few individuals of Blenniidae and Gobiidae recorded in Corsica-May were more abundant in forest (Table 3).

## Discussion

Taxonomic diversity and total density of small-medium resident fish were highest in *Cystoseira* forest and lowest in barren. Turf showed intermediate values, but this finding should be regarded with caution as turf was sampled only at 4 sites in Menorca. Total fish biomass did not differ between habitats because the larger average size of fish in barrens compensated for their lower densities. Effects of habitat were consistent between regions-times in terms of direction, but showed variability in their magnitude. This was mainly due to high densities of very small new settlers of Gobiesocidae, Clinidae, Blenniidae, Gobiidae, *Coris julis* and *Symphodus* spp. in *Cystoseira* forests of Menorca-July. This suggests that *Cystoseira* forests, at least in Menorca, act as nursery habitat for these species, as previously suggested for *Symphodus* spp. in Corsica [40]. Settler densities were considerably lower at Corsica-May probably due to the sampling period (*i*.*e*. May) that was too early to detect settlement peaks for these species (from late spring to autumn [30, 40, 65–68]). Therefore, observed difference between regions were likely due, at least in part, to seasonal variability.

Small-medium resident fish were also more diverse in *Cystoseira* forests in terms of trophic groups. Fish with the highest densities in *Cystoseira* forests included both juvenile and adult fish belonging to (1) the CB taxa Blenniidae, Gobiidae, Trypterigiidae and Cliniidae, which are omnivores, micro- or meso- carnivorous (depending on taxa and/or life stage), (2) the NB Labridae, which are mainly mesocarnivorous, and (3) the CB Scorpaenidae and NB Serranidae, which are meso- or macro- carnivorous (depending on life stages) whose food items include small-sized CB and/or NB fish [30, 69, 70]. In contrast, the only fishes that displayed the highest densities in barrens were the NB sea urchin feeders *Diplodus* spp. and *Thalassoma pavo* [28] (See S2 Text for more details).

Small-sized fish and large-sized macrocarnivorous fish cohabit at higher densities in *Cystoseira* forests. This may be due to lower mortality in *Cystoseira* forest (starvation- and/or predation- induced) and/or net immigration from other habitats (due to habitat selection toward *Cystoseira* forests) [71]. Lower mortality and habitat selection could both be related to: 1) increased food resources in *Cystoseira* forest (invertebrates for small-sized fish, and invertebrates and small-sized fish for macrocarnivorous fish [72, 73]) and/or 2) forest structural complexity providing shelters against predators (small sized-fish threatened by macrocarnivorous fish, and macrocarnivorous fish threatened by higher order predators such as *Epinephelus marginatus* or *Dentex dentex* [71]).

CB fish included omnivorous and micro-carnivorous fish while NB fish did not. Considering their relatively high densities (92% of small-medium resident fish density) and their exclusiveness at intermediate trophic positions (among small-medium resident fish), CB fish may play a crucial role with regard to the trophic functioning of *Cystoseira* forest-dominated ecosystems. This highlights the need for further research on CB fish assemblages, as they are underestimated by UVC sampling [46] and therefore have been largely understudied. As demonstrated in this study, EASV is an effective quantitative harvesting method that can be used in complex habitats to study CB assemblages.

Our finding of higher diversity and densities of small-medium resident fish in *Cystoseira* forest than in barren corroborates broad patterns of higher fish diversity and density in high complexity biotic habitats compared to adjacent, structurally less complex habitats. Examples include seagrass meadows compared to adjacent bare sediments [74–78], mangrove roots compared to adjacent mud flats [79–81], algal forests [59, 82] or other erect or turf-forming algae [27] compared to adjacent bare rocks. Studies that compared some components of the whole fish assemblage between *Cystoseira* forest and various habitat types [33–36, 40] also reported higher fish diversity and/or density and/or biomass in *Cystoseira* forests, at least for some fish taxa and/or at some of their life stages (see S2 Text). Although all the studies assessed potential variability using the same variables (*i*.*e*. diversity, density, biomass), the response of each single variable was inconsistent among the studies. Such discrepancies among studies (including ours) may be primarily related to: (1) differences in the fish assemblages studied, which were either the whole NB fish assemblage including transient and large resident fish [33–35], only NB juvenile fish [40], or the assemblage of small-medium resident CB and NB fish (the present study), and/or (2) the inaccurate sampling of CB fish using UVC [36], and/or (3) sampling designs where the results are confounded with protection levels [33] or abiotic variations [34–36].

Our study has significantly contributed to the knowledge of CB and NB small-medium resident fish assemblage structure in *Cystoseira* forests and barrens (and turf to some extent). Based on the differences between habitat types, we can speculate that *Cystoseira* forest degradation into barrens (and likely turfs) may reduce the density and diversity of small-medium resident fish. This includes both juvenile fish which are important for population replenishment as well as socio-economically important fish species such as *Scorpaena* spp. and *Serranus* spp. However, we note that our evidence relates to a relatively short observational period (May or July) in two different localities and that our findings are based on a space-for-time approach that does not allow consideration of all alternative hypotheses. A manipulative approach would enable a broader range of inference, but would need to include large-scale removal experiments of *Cystoseira* spp, which may not be appropriate given its conservation status. Hence, to better assess the community-wide impact of *Cystoseira* forest losses, it is crucial to set up long-term monitoring of Mediterranean subtidal macroalgal habitats and associated communities [19]. In addition, further space-for-time studies would be of value (1) to compare the assemblage structure of large and/or highly mobile NB fish between habitats and (2) to extend sampling locations and periods (all seasons during several years) in order to draw a more robust picture of the impact of changes in marine vegetation on fish assemblage structure.

## Supporting Information

S1 Fig Sampling of necto-benthic fish assemblage structure using the UVC methodology ‘9 m² stationary-point snapshot-count’. The 9m² sampling area was the semicircle 2.5 m in radius in front of the diver, without considering the inner part, semicircle 0.7 m in radius.
(TIFF)

S2 Fig Fish body-size distributions compared across habitat types and Localities. Curves are smoothed histograms (Kernel density estimations) of total lengths of all sampled fish (crypto- or necto- benthic) within each level of the combined factor habitat X locality-protection (region-time). The surfaces below the curves (the integrals) are proportional to fish densities (abundance per sampling unit). Total length distributions are also presented using Tukey's boxplots. (See also S1 Text). Modified from Thiriet *et al*. [30].
(TIFF)

S1 Table List of the macrophytes operational taxonomic units and their functional group. (DOCX)

S2 Table Geographical coordinates of the 23 sampling sites. Latitude (North) and Longitude (East) are in decimal degrees (See also Fig 2).
(DOCX)

S1 Text Supplementary analysis of fish body size distributions across habitats: Methods and Results. (See also S2 Fig)
(DOCX)

S2 Text Possible mechanisms underlying differences in fish assemblage composition between *Cystoseira* forest, turfs and barrens. A discussion enriched by previous studies on density patterns and fish life history traits found in the literature.
(DOCX)
