# Supplementary figures and images for: Abundance and Diversity of Crypto- and Necto-Benthic Coastal Fish Are Higher in Marine Forests than in Structurally Less Complex Macroalgal Assemblages
Source: PLoS One. 2016 Oct 19;11(10):e0164121. doi: 10.1371/journal.pone.0164121 (PMC5070871; doi:10.1371/journal.pone.0164121)

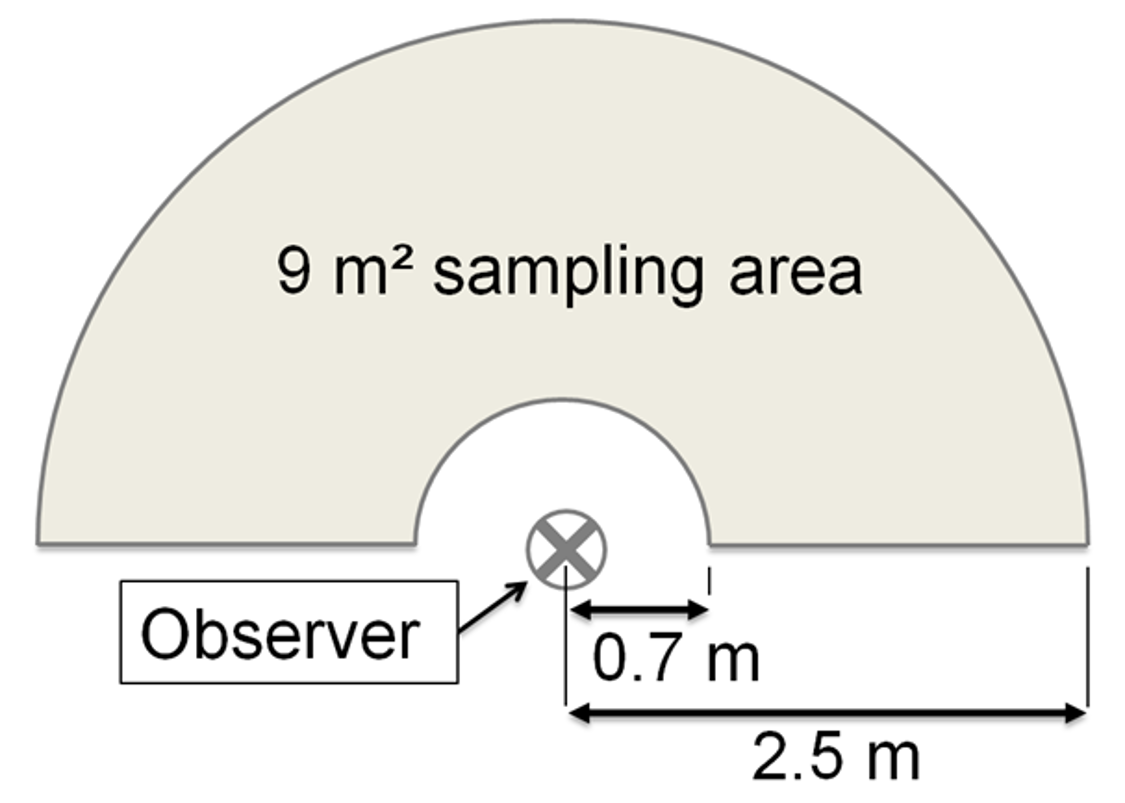

Supplement: S1 Fig — The 9m² sampling area was the semicircle 2.5 m in radius in front of the diver, without considering the inner part, semicircle 0.7 m in radius. (TIFF) [file pone.0164121.s001.tiff]

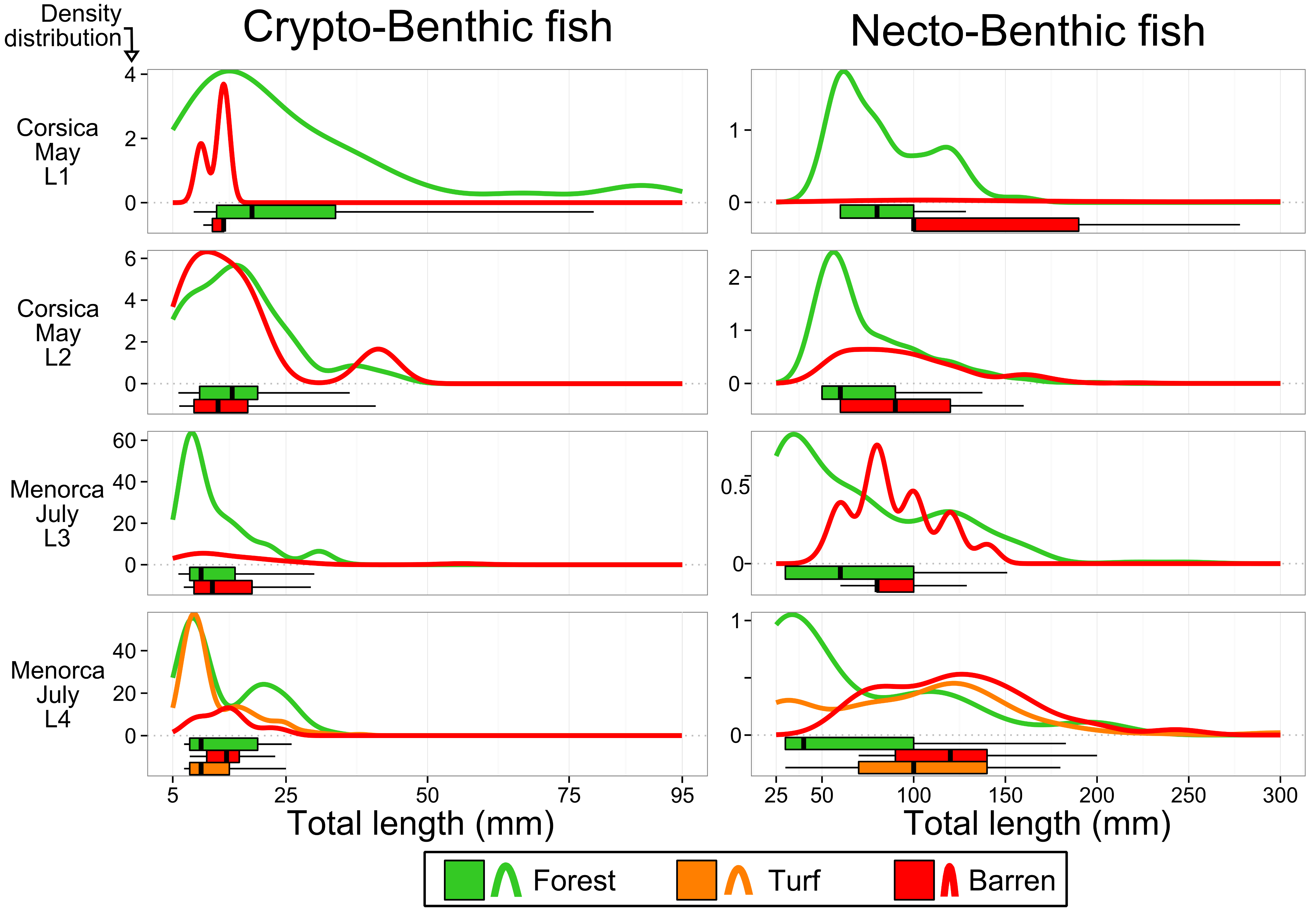

Supplement: S2 Fig — Curves are smoothed histograms (Kernel density estimations) of total lengths of all sampled fish (crypto- or necto- benthic) within each level of the combined factor habitat X locality-protection (region-time). The surfaces below the curves (the integrals) are proportional to fish densities (abundance per sampling unit). Total length distributions are also presented using Tukey's boxplots. (See also S1 Text). Modified from Thiriet et al. [30]. (TIFF) [file pone.0164121.s002.tiff]
