# Supplementary material for: Abundance and Diversity of Crypto- and Necto-Benthic Coastal Fish Are Higher in Marine Forests than in Structurally Less Complex Macroalgal Assemblages
Source: PLoS One. 2016 Oct 19;11(10):e0164121. doi: 10.1371/journal.pone.0164121 (PMC5070871; doi:10.1371/journal.pone.0164121)
Supplement: S1 Table — (DOCX) [file pone.0164121.s003.docx]

| S1 Table. List of the macrophyte operational taxonomic units and their functional group | |
| --- | --- |
| **Operational taxonomic units** | **Functional groups** |
| *Cystoseira balearica* | Canopy-forming Algae |
| *Cystoseira compressa* | Canopy-forming Algae |
| *Sargassum* spp. | Canopy-forming Algae |
| *Dictyota* spp. | Large erect algae |
| *Halopteris scoparia* | Large erect algae |
| *Padina pavonica* | Large erect algae |
| *Dictyopteris polypodioides* | Large erect algae |
| *Digenea simplex* | Large erect algae |
| *Acetabularia acetabulum* | Small erect algae |
| *Anadyomene stellata* | Small erect algae |
| *Botryocladia* spp. | Small erect algae |
| *Caulerpa racemosa* | Small erect algae |
| *Dasycladus vermicularis* | Small erect algae |
| *Flabellia petiolata* | Small erect algae |
| *Halimeda tuna* | Small erect algae |
| *Valonia* sp. | Small erect algae |
| *Amphiroa rigida* | Turf-forming articulated Corallinales |
| *Corallina elongata* | Turf-forming articulated Corallinales |
| *Jania rubens* | Turf-forming articulated Corallinales |
| Other articulated Corallinales | Turf-forming articulated Corallinales |
| *Laurencia* complex | Turf-forming filamentous algae |
| Other green and red filamentous algae | Turf-forming filamentous algae |
| *Codium bursa* | Massive algae |
