# Supplementary material for: Abundance and Diversity of Crypto- and Necto-Benthic Coastal Fish Are Higher in Marine Forests than in Structurally Less Complex Macroalgal Assemblages
Source: PLoS One. 2016 Oct 19;11(10):e0164121. doi: 10.1371/journal.pone.0164121 (PMC5070871; doi:10.1371/journal.pone.0164121)
Supplement: S2 Table — Latitude (North) and Longitude (East) are in decimal degrees (See also Fig 2). (DOCX) [file pone.0164121.s004.docx]

| **S2 Table. Geographical coordinates of the 23 sampling sites**. Latitude (North) and Longitude (East) are in decimal degrees. | | | | | |
| --- | --- | --- | --- | --- | --- |
| **Region** | **Locality** | **Site** | **Habitat-type** | **Latitude** | **Longitude** |
| Corsica | L1 | S01 | Forest | 42.349419° N | 8.551950° E |
| Corsica | L1 | S02 | Barren | 42.349428° N | 8.551378° E |
| Corsica | L1 | S03 | Forest | 42.349506° N | 8.551128° E |
| Corsica | L1 | S04 | Barren | 42.349667° N | 8.551906° E |
| Corsica | L2 | S05 | Barren | 42.563431° N | 8.715969° E |
| Corsica | L2 | S06 | Forest | 42.565281° N | 8.709889° E |
| Corsica | L2 | S07 | Forest | 42.568344° N | 8.744019° E |
| Corsica | L2 | S08 | Barren | 42.570447° N | 8.757133° E |
| Corsica | L2 | S09 | Barren | 42.567611° N | 8.762692° E |
| Corsica | L2 | S10 | Forest | 42.585544° N | 8.800311° E |
| Menorca | L3 | S11 | Forest | 40.057113° N | 3.989736° E |
| Menorca | L3 | S12 | Forest | 40.057244° N | 3.990075° E |
| Menorca | L3 | S13 | Barren | 40.057467° N | 3.990739° E |
| Menorca | L3 | S14 | Barren | 40.057598° N | 3.991253° E |
| Menorca | L3 | S15 | Forest | 40.059603° N | 4.000748° E |
| Menorca | L3 | S16 | Forest | 40.059753° N | 4.001544° E |
| Menorca | L4 | S17 | Barren | 40.059781° N | 4.172583° E |
| Menorca | L4 | S18 | Barren | 40.058035° N | 4.172475° E |
| Menorca | L4 | S19 | Turf | 40.036000° N | 4.166075° E |
| Menorca | L4 | S20 | Turf | 40.036647° N | 4.167068° E |
| Menorca | L4 | S21 | Turf | 40.035773° N | 4.172443° E |
| Menorca | L4 | S22 | Turf | 40.035094° N | 4.173308° E |
| Menorca | L4 | S23 | Forest | 40.034739° N | 4.173472° E |
