# Supplementary material for: Abundance and Diversity of Crypto- and Necto-Benthic Coastal Fish Are Higher in Marine Forests than in Structurally Less Complex Macroalgal Assemblages
Source: PLoS One. 2016 Oct 19;11(10):e0164121. doi: 10.1371/journal.pone.0164121 (PMC5070871; doi:10.1371/journal.pone.0164121)
Supplement: S1 Text — (See also S2 Fig) (DOCX) [file pone.0164121.s005.docx]

**S1 Text. Supplementary analysis of fish body size distributions across habitats: Methods and Results.** (see also Fig S2)

# Aim of the supplementary analysis

Body-size (total length) distributions of Crypto-benthic and Necto-Benthic fish individuals were visualized in order to (1) better understand their relative contribution to total density and total biomass, (2) visualize putative differences among habitats.

# Methods

A smoothed histogram (Kernel Density Estimation) was plotted for each level of combinations of habitat x locality-Protection (region-time). For each histogram, the Kernel Density Estimation was computed on body-size of all fish individuals pooled. At this stage, the surface below the Kernel Density curve equals 1. Kernel Density Estimations were multiplied by the total fish abundance averaged over sample, in order to plot the count curve of which the surface area below represents the averaged fish density. This was performed in R Environment [[1](#_ENREF_1)] using the library ggplot2 [[2](#_ENREF_2)].

# Results

Concerning the differences in body-sizes between crypto-benthic and necto-benthic fish, most of crypto-benthic fish were smaller than necto-benthic fish regardless of the habitats (Fig S2). Specifically, more than 75% of crypto-benthic fish individuals were smaller than 25 mm (3rd quartile < 25 mm in boxplots Fig S2), excluding the forest sites of the locality Scandola (Corsica-May) where the 3rd quartile was close to 35mm due to the presence of some larger individuals (until 95mm). In contrast, all necto-benthic fish individuals were larger than 25 mm. More than 75% of necto-benthic fish individuals were larger than 50 mm, excluding the forest sites of Menorca-July where a high proportion of fish sized between 25 and 50 mm was recorded. On the basis of the positive correlation between body-weight and body-size, the overall larger sizes of necto-benthic fish explained the dominance of necto-benthic fish in terms of biomass despite the dominance of crypto-benthic fish in terms of densities (see Fig 5).

With regard to differences between habitats in crypto-benthic fish body-size distributions, patterns were not consistent between the 4 localities. This was mainly due to high variability among barrens compared to the relative consistency among forests. Nevertheless, in 3 out 4 localities (excluding L2), the curve (smoothed histogram indicating fish density as a function of their body size, Fig S2) of forest never crossed the curve of barren and was always above. This indicated that fish were more abundant in forest, regardless of their body size. In the fourth locality, Calvi, curves of forest and barren followed the same trend indicating that density (see Fig 6) and body-size were similar. Focusing on locality L4 (the only one where it was possible to sample the turf), the turf curve was superimposed on the forest curve from 5 to 15 mm and was below further on, due to a second mode in the forest curve around 20 mm. This indicated that very small individuals (5 to 15 mm) are as abundant in forest as in turf, while larger individuals (especially those sized around 20 mm) were more abundant in forest.

Concerning differences between habitats in necto-benthic fish body-size distributions, patterns were also inconsistent between region-times. In Corsica-May, the forest curve was above the barren curve in both locality, indicating higher fish densities of all sizes in forests. In Menorca-July, the forest curve was above the barren curve from 25 to 70 mm, indicating higher densities of small-sized (25 to 70 mm) fish in forest. From 70 mm, trends were different between the two locality. In North, fish sized between 70 and 120 mm were more abundant in barren, and fish between 120 mm and 200 mm were slightly more abundant in forest. In East, fish larger than 120 mm (up to the largest observed at 300 mm) were more abundant in barren (especially those sized around 130 mm). Concerning turf (only in East), densities of fish sized between 25 and 70 mm were intermediate between the lowest of barren and the highest of forest. From 70 mm, size distribution of turf curve followed the same trend as barren (similar curve shape) but indicated lesser densities (curve positioned below).

In summary, the higher fish densities in forest compared to barren were mainly driven by small fish individuals that were more abundant in forest than in barren (especially for necto-benthic fish). The higher densities of crypto-benthic fish in forest of Menorca-July compared to forest of Corsica-May were mainly due to the very-small fish individuals that were highly abundant in forests of Menorca-July.

# References

1. R Development Core Team. R: A Language and Environment for Statistical Computing. 2013.

2. Wickham H. ggplot2: elegant graphics for data analysis: Springer New York; 2009.
