# Supplementary material for: Abundance and Diversity of Crypto- and Necto-Benthic Coastal Fish Are Higher in Marine Forests than in Structurally Less Complex Macroalgal Assemblages
Source: PLoS One. 2016 Oct 19;11(10):e0164121. doi: 10.1371/journal.pone.0164121 (PMC5070871; doi:10.1371/journal.pone.0164121)
Supplement: S2 Text — A discussion enriched by previous studies on density patterns and fish life history traits found in the literature. (DOCX) [file pone.0164121.s006.docx]

S2 Text. Possible mechanisms underlying differences in fish assemblage composition between *Cystoseira* forest, turfs and barrens.

The association of some fish taxa (or of some life history stages of taxa, such as juvenile stage) with either *Cystoseira* forests, turfs or barrens (See Results section, Table 3 and Fig. 7), may depend upon their life history traits such as feeding habits and morphological and behavioral anti-predation strategies. We present here some possible hypotheses regarding the possible factors that determined the observed taxa-specific density patterns, supplemented by their life history traits found in the literature.

# Fish associated with *Cystoseira* forests

Fish taxa that were more abundant (or only present) in forests compared to turf and barren consistently across regions-times were taxonomically and functionally diversified. They included the crypto-benthic fish Trypterygidae [[diurnal, eggs and invertebrates feeders, 1](#_ENREF_1)], Scorpaeniade [[nocturnal sit-and-wait macrocarnivores, invertebrates and fish feeders, 2](#_ENREF_2)] and *Serranus cabrilla* crypto-benthic juveniles, and the necto-benthic fish *Serranus* sp. (sub)-adults [diurnal stalck-and-attack macrocarnivores, invertebrates and fish feeders,[1](#_ENREF_1), [3](#_ENREF_3), [4](#_ENREF_4)], *Symphodus roissali* and *S. ocellatus* [[diurnal invertebrates feeders, 1](#_ENREF_1)]. The ubiquitous *Coris julis* [diurnal invertebrates -sea urchins included- feeders, [1](#_ENREF_1), [5](#_ENREF_5)] was also present in high densities.

These density patterns observed for *Symphodus* spp. juveniles and adults are consistent with previous studies on *Cystoseira* forest [[6](#_ENREF_6), [7](#_ENREF_7)]. Other studies also highlighted Trypterygidae and *Symphodus* spp's preference for habitats with erect macrophytes in regions where *Cystoseira* forest were not present [[8-11](#_ENREF_8)]. In contrast, the currently observed higher densities of *Serranus* spp. in forests were more surprising, since some studies found that their densities were not affected by the structure of the macrophyte assemblage [[7](#_ENREF_7), [12](#_ENREF_12)].

The coexistence of both small (prey) fish and macrocarnivores (in part piscivores) fish in the *Cystoseira* forest might be due to lower (starvation- and/or predation- induced) mortality in forest and/or habitat selection (immigration). However, the association of prey fish with the predator-rich forest might be related to the fact that prey may escape predation because of the forest canopy (authors’ unpublished data), which may outweigh the higher (compared to barren) encounter rates with predators [[13](#_ENREF_13)]. Higher densities of their food resources (invertebrates) in *Cystoseira* forest compared to barren [[14](#_ENREF_14), [15](#_ENREF_15)] may be also a determining factor. Macrocarnivores association to forest, on the other hand, may be linked to higher prey densities. Both invertebrates [[14](#_ENREF_14), [15](#_ENREF_15)] and small-sized fish [[7, the present study](#_ENREF_7)] are more abundant in *Cystoseira* forests. This may outweigh the possible negative effect of habitat structure on macrocarnivores' foraging efficiency [[16](#_ENREF_16)]. Moreover, foraging strategy that involves almost no predator movements, such as sit-and-wait (and stalk-and-attack in a lesser extent), may be unaffected by habitat structure [[17](#_ENREF_17)] or may be facilitated [[18](#_ENREF_18)]. Lastly, macrocarnivores may be prey of higher order predators such as *E. marginatus* and *D. dentex*. From this perspective, the structural complexity provided by *Cystoseira* sp. may reduce the intensity of prey-predator lethal interactions at every trophic level (at least a few individuals may always survive) and consequently promote the persistence of populations forming a diversified faunal community associated with the *Cystoseira* forest [[19](#_ENREF_19)].

# Fish associated with barren

The only taxa showing higher densities in barren than in forest were *Diplodus sargus* (in Corsica-May), *D. vulgaris* and *Thalassoma pavo* (in Menorca-july). The ubiquitous *Coris julis* (NBJ excluded) was also present in high densities in barren in both regions-times. These species are the 4 most important seaurchin feeders in Mediterranean rocky reefs [[5 and references therein](#_ENREF_5)]. Their presence in barrens (that are maintained by sea urchins) might be linked to foraging activity. However, their densities were probably insufficient for controlling the sea urchin population and allowing recovery from barren state [[25](#_ENREF_25)]. Concerning the non-dependence of these larger taxa upon macrophyte refuges, this might be related to their larger body-size (compared to fish associated with forest) that provides relative safety against predation [['refuge in size', e.g. 26](#_ENREF_26)].

Concerning the few Blenniidae and Gobiidae recorded in barren compared to forest, their presence may be due to the presence of holes in the substrate or some clumps of macrophytes that could have provided sufficient shelter. Parravicini *et al.* [[27](#_ENREF_27)] evidenced a positive correlation between the number of holes and densities of Blenniidae. Kovačić et al [[28](#_ENREF_28)] also recorded some Blenniidae and Gobiidae species associated with bare rocks. He defined these species as 'epi-benthic' since they were not hidden below or within a physical structure, but rather used crypsis with susbtrate.

# Fish associated with turf

Our data in Menorca-July suggested that turf hosted (1) new settlers - but not adults - of Blenniidae and Gobiidae, in very high densities (as in forests, and more than in barrens), and (2) the 4 sea urchin feeders in intermediate densities (more than in forests and less than in barrens). The first pattern might be related to the very high structural complexity of the turf layer (providing only very small interstitial spaces), which might be accessible to the very small sized settlers that seek shelter, but not accessible to adults, which might explain their absence in turf. In contrsast, the fact that turfs hosted in intermediate densities the large-sized urchin feeders was probably not related to the impossibility of hiding (since it is also impossible to hide in barren where they were more abundant), but rather due to lower food resources than in barren (or at least lower accessibility to resources due to physical constraints, [[29](#_ENREF_29)]).

# References:

1. Stergiou KI, Karpouzi VS. Feeding habits and trophic levels of Mediterranean fish. Reviews in Fish Biology and Fisheries. 2002;11(3):217-54. doi: 10.1023/a:1020556722822.

2. Harmelin-Vivien ML, Kaim-Malka RA, Ledoyer M, Jacob-Abraham SS. Food partitioning among scorpaenid fishes in Mediterranean seagrass beds. Journal of Fish Biology. 1989;34(5):715-34. doi: 10.1111/j.1095-8649.1989.tb03352.x.

3. Alos J, March D, Palmer M, Grau A, Morales-Nin B. Spatial and temporal patterns in Serranus cabrilla habitat use in the NW Mediterranean revealed by acoustic telemetry. Marine Ecology Progress Series. 2011;427:173-86. doi: 10.3354/meps09042.

4. Viladiu C, Vandewalle P, Osse JWM, Casinos A. Suction Feeding Strategies of Two Species of Mediterranean Serranidae (Serranus Cabrilla and Serranus Scriba). Netherlands Journal of Zoology. 1999;49(2):81-95. doi: 10.1163/156854299x00065.

5. Guidetti P. Predator diversity and density affect levels of predation upon strongly interactive species in temperate rocky reefs. Oecologia. 2007;154(3):513-20. doi: 10.1007/s00442-007-0845-5. PubMed PMID: ISI:000251146900008.

6. Giakoumi S, Kokkoris GD. Effects of habitat and substrate complexity on shallow sublittoral fish assemblages in the Cyclades Archipelago, North-eastern Mediterranean sea. Mediterranean Marine Science. 2013;14(1):58-68. PubMed PMID: WOS:000320294500008.

7. Cheminée A, Sala E, Pastor J, Bodilis P, Thiriet P, Mangialajo L, et al. Nursery value of Cystoseira forests for Mediterranean rocky reef fishes. Journal of Experimental Marine Biology and Ecology. 2013;442(0):70-9. doi: http://dx.doi.org/10.1016/j.jembe.2013.02.003.

8. Ruitton S, Francour P, Boudouresque CF. Relationships between Algae, Benthic Herbivorous Invertebrates and Fishes in Rocky Sublittoral Communities of a Temperate Sea (Mediterranean). Estuarine, Coastal and Shelf Science. 2000;50(2):217-30.

9. Letourneur Y, Ruitton S, Sartoretto S. Environmental and benthic habitat factors structuring the spatial distribution of a summer infralittoral fish assemblage in the north-western Mediterranean Sea. Journal of the Marine Biological Association of the United Kingdom. 2003;83(1):193-204. PubMed PMID: ISI:000181637500026.

10. Garcia Rubies A, Macpherson E. Substrate use and temporal pattern of recruitment in juvenile fishes of the mediterranean littoral. Marine Biology. 1995;124(1):35-42. doi: 10.1007/bf00349144. PubMed PMID: WOS:A1995TH40300005.

11. La Mesa G, Micalizzi M, Giaccone G, Vacchi M. Cryptobenthic fishes of the “Ciclopi Islands” marine reserve (central Mediterranean Sea): assemblage composition, structure and relations with habitat features. Marine Biology. 2004;145(2):233-42. doi: 10.1007/s00227-004-1315-9.

12. La Mesa GL, Louisy P, Vacchi M. Assessment of microhabitat preferences in juveniles dusky grouper (Epinephelus marginatus) by visual sampling. Marine Biology. 2002;140:175-85. doi: citeulike-article-id:195235.

13. Lima SL. Strong preferences for apparently dangerous habitats - a consequence of differential escape from predators. Oikos. 1992;64(3):597-600. doi: 10.2307/3545181. PubMed PMID: WOS:A1992JG51000023.

14. Gozler AM, Kopuz U, Agirbas E. Seasonal changes of invertebrate fauna associated with Cystoseira barbata facies of Southeastern Black Sea coast. African Journal of Biotechnology. 2010;9(51):8852-9. PubMed PMID: ISI:000285846100020.

15. Chemello R, Milazzo M. Effect of algal architecture on associated fauna: some evidence from phytal molluscs. Marine Biology. 2002;140(5):981-90. doi: 10.1007/s00227-002-0777-x. PubMed PMID: WOS:000176211500010.

16. Lannin R, Hovel K. Variable prey density modifies the effects of seagrass habitat structure on predator&#x2212;prey interactions. Marine Ecology Progress Series. 2011;442:59-70. doi: 10.3354/meps09393.

17. Schultz S, Kruschel C. Frequency and success of ambush and chase predation in fish assemblages associated with seagrass and bare sediment in an Adriatic lagoon. Hydrobiologia. 2010;649(1):25-37. doi: 10.1007/s10750-010-0256-1.

18. Rilov G, Figueira WF, Lyman SJ, Crowder LB. Complex habitats may not always benefit prey: linking visual field with reef fish behavior and distribution. Marine Ecology-Progress Series. 2007;329:225-38. doi: 10.3354/meps329225. PubMed PMID: WOS:000245319700019.

19. Janssen A, Sabelis MW, Magalhaes S, Montserrat M, Van der Hammen T. Habitat structure affects intraguild predation. Ecology. 2007;88(11):2713-9. doi: 10.1890/06-1408.1. PubMed PMID: WOS:000251067900006.

20. Beldade R, Erzini K, Goncalves EJ. Composition and temporal dynamics of a temperate rocky cryptobenthic fish assemblage. Journal of the Marine Biological Association of the United Kingdom. 2006;86(5):1221-8. doi: 10.1017/s0025315406014226. PubMed PMID: WOS:000241311000035.

21. Felix-Hackradt FC, Hackradt CW, Trevino-Oton J, Segovia-Viadero M, Perez-Ruzafa A, Garcia-Charton JA. Environmental determinants on fish post-larval distribution in coastal areas of south-western Mediterranean Sea. Estuar Coast Shelf Sci. 2013;129:59-72. doi: 10.1016/j.ecss.2013.05.029. PubMed PMID: WOS:000323361400008.

22. Raventos N, Macpherson E. Environmental influences on temporal patterns of settlement in two littoral labrid fishes in the Mediterranean Sea. Estuar Coast Shelf Sci. 2005;63(4):479-87. doi: 10.1016/j.ecss.2004.11.018. PubMed PMID: WOS:000229809900002.

23. Lejeune P. Etude écoéthologique des comportements reproducteurs et sociaux des Labridae méditerranéens des genres Symphodus (Rafinesque 1810) et Coris (Lacepede 1802). Cahiers d’Ethologie Appliquée. 1985; 5:1–208.

24. Thiriet P. Comparaison de la structure des peuplements de poissons et des processus écologiques sous-jacents, entre les forêts de Cystoseires et des habitats structurellement moins complexes, dans l'Infralittoral rocheux de Méditerranée nord-occidentale [PhD thesis]. PhD Thesis: University Nice Sophia Antipolis - EA 4228 ECOMERS; 2014.

25. Guidetti P, Sala E. Community-wide effects of marine reserves in the Mediterranean Sea. Marine Ecology-Progress Series. 2007;335:43-56. PubMed PMID: ISI:000246486800004.

26. Olson M. Predator-prey interactions in size-structured fish communities: implications of prey growth. Oecologia. 1996;108(4):757-63. doi: 10.1007/bf00329052.

27. Parravicini V, Donato M, Morri C, Villa E, Bianchi CN. Date mussel harvesting favours some blennioids. Journal of Fish Biology. 2008;73(10):2371-9. doi: 10.1111/j.1095-8649.2008.02085.x.

28. Kovačić M, Patzner RA, Schliewen U. A first quantitative assessment of the ecology of cryptobenthic fishes in the Mediterranean Sea. Marine Biology. 2012;159(12):2731-42. doi: 10.1007/s00227-012-2030-6.

29. Thiriet P, Cheminée A, Mangialajo L, Francour P. How 3D Complexity of Macrophyte-Formed Habitats Affect the Processes Structuring Fish Assemblages Within Coastal Temperate Seascapes? In: Musard O, Le Dû-Blayo L, Francour P, Beurier J-P, Feunteun E, Talassinos L, editors. Underwater Seascapes: Springer International Publishing; 2014. p. 185-99.
